# Biological and molecular characterization of *Aromia bungii* (Faldermann, 1835) (Coleoptera: Cerambycidae), an emerging pest of stone fruits in Europe

**DOI:** 10.1038/s41598-020-63959-9

**Published:** 2020-04-28

**Authors:** Elia Russo, Francesco Nugnes, Francesco Vicinanza, Antonio P. Garonna, Umberto Bernardo

**Affiliations:** 1 CNR, Institute for Sustainable Plant Protection, Portici, Italy; 2 Department of Agricultural Sciences, University of Napoli “Federico II”, Section BiPAF, Portici, Italy

**Keywords:** Invasive species, Haplotypes, Molecular biology, Entomology, Molecular ecology

## Abstract

The red-necked longhorn beetle (RLB) *Aromia bungii* (Fald.) is an emerging pest of stone fruit trees, native to East Asia, accidentally introduced in Europe (Germany and Italy) and Japan. Threatening seriously the stone fruit crops in Europe, RLB was added to both the EPPO A1 and priority pest lists of quarantine species. Molecular analyses highlighted that all specimens recovered in southern Italy share the same haplotype, different from the German one, supporting that the invasive process in Europe started from at least two independent introductions. To fill the existing gap of biological knowledge about *A*. *bungii*, several laboratory tests were carried out on specimens collected in the outbreak area of Naples (Italy). Results suggest a high biotic potential of the RLB Italian population. Females showed a short pre-oviposition period while the period of oviposition lasted about three weeks, with a rate of 24.2 eggs/day. Each female laid an average of 587.5 eggs and spawned the largest amount of eggs during the first week after emergence. Fed males live up to 62 days at 20 °C while fed females about 63 days at 25 °C. These results are crucial to draw up a multi-facet IPM approach against *A*. *bungii* in the outbreak areas.

## Introduction

The growing international trade of living plants and plant-based products increases the likelihood of introducing alien organisms to new areas, where they may develop as invasive pests. Italy is particularly troubled by this phenomenon because of its geographical position and habitat patterns favourable to the spread and the establishment of allochthonous insect species^1^, with dramatic economic and ecological impacts to agricultural and natural forested areas^2–4^.

The red-necked longhorn beetle (RLB) *Aromia bungii* (Faldermann, 1835) (Coleoptera: Cerambycidae) is an invasive wood-borer pest of *Prunus* trees, including important commercial varieties. *Aromia bungii* belongs to the subfamily Cerambycinae, tribes Callichromatini, and it is native to Eastern Asia (China, Korea, Taiwan, Vietnam and Mongolia)^5,6^, where it is considered the major pest of stone fruit trees such as peach, apricot, plum and cherry^5,7^.

The first field records of this pest outside its native area date back to 2011, when RLB was found in Bavaria (Germany) on *Prunus domestica* subsp. *insititia*^8^. A year later, as a result of an important outbreak found in Southern Italy, *A*. *bungii* was added to the EPPO Alert List^9^. Its presence was officially reported after the record of massive infestations that affected different host species (*P*. *armeniaca*, *P*. *domestica*, and *P*. *avium*), both in commercial orchards and in private gardens located in Naples and in a few neighbouring municipalities. In 2013, a new outbreak was recorded in Northern Italy near Milan^10^ leading to the inclusion of RLB in the A1 list of quarantine pests^11^. Because the presence of *A*. *bungii* is known in a limited area of the European Union and due to its more serious economic, environmental, and social impacts than other harmful quarantine organisms, it has been recently included in the priority list of relevant quarantine pests to the Union territory^12^.

*Aromia bungii* is currently established in an area of about 250 km^2^ in the Campania Region and is the focus of an eradication effort there^13^. The pest is in a containment state in Northern Italy^14,15^, while in Germany it is considered as transient, under eradication^16,17^. *Aromia bungii* has also been reported in Japan^6,18^ where it appears to be spreading rapidly, threatening the cherry blossom trees across the country^6,19,20^. Wood packaging material or wooden products infested by immatures might be behind the accidental introduction of RLB in Europe and Japan^21^ as reported for other invasive longhorn beetles^22,23^.

Although most longhorn beetles develop on dead or decaying trees, *A*. *bungii* develops on healthy host plants, infesting the trunks and main branches^5,24^. Infested trees may be easily identified by the presence of abundant frass at the tree base and exit holes on the bark^5,24–27^. The life cycle lasts 1–4 years showing a clear latitudinal gradient^5,25,28^.

As a result of the new pest status of RLB, recent research has shed some light on morphological structures, flight behaviour, semiochemicals, intraspecific communication, mate location, and natural control^29–34^. In China, authors investigated mainly *A*. *bungii* distribution, life cycle, bionomics, injury levels, and control options^25,26,28,35^. Nevertheless, its biology and genetic variability are not well known, and some reproductive traits, such as lifetime fecundity, are unexplored^21^. Knowledge of these parameters is essential to assess the potential damage of the invasive pest, to develop methods of survey and monitoring, and the screening of management options of RLB in the invaded countries. Our objectives were to genetically characterize the *A*. *bungii* population in southern Italy, and evaluate its reproductive patterns and biological traits under laboratory conditions.

## Results

### Collection of RLB adults from infested logs

A total of 310 adult individuals of *A*. *bungii* emerged in entomological cages (156 females and 154 males, sex ratio 0.5) at 25 ± 1 °C. The first adults emerged on 24 April and emergence continued until 9 July (77 days). All specimens used in the tests were measured and results are summarized in Table 1. Body length was chosen as an indicator of body size to verify correlations with biological variables, because it was related to metatibia (r = 0.947, F = 2783.03; df = 1, 156; *P* < 0.00001) and elytral length (r = 0.971, F = 5201.49; df = 1, 156; *P* < 0.00001).

**Table 1:** Size of *A*. *bungii* adults in mm (mean ± SE) with the range of variation in round brackets. In square brackets is the type of experiments that measured specimens were used for.

Sex [test]	Body length	Elytra	Metatibia
Male (n = 60)	28.4 ± 0.42 (17-36)	17.4 ± 0.28 (9-22.5)	10.5 ± 0.15 (6–13)
[Longevity]			
Female (n = 60)	31.5 ± 0.46 (20–40)	19.9 ± 0.27 (13–25)	11.3 ± 0.17 (8–15)
[Longevity]			
Female (n = 10)	29.8 ± 0.49 (27–31)	20.5 ± 0.40 (18.5–22)	10.9 ± 0.35 (9–12)
[Dissected]			
Female (n = 18)	30.3 ± 0.54 (24–35)	18.7 ± 0.44 (13–21)	10.4 ± 0.26 (9–13)
[Fecundity]			

### Morphological and molecular identification

COI sequencing revealed that all the *A*. *bungii* samples collected in Campania shared the same mitochondrial haplotype (Table 2). Blast search showed that the haplotype found in Campania had five and two mismatches with sequences from China (KF737790) and Germany (KM443233), respectively. The other sequence of *A*. *bungii* from China available in GenBank (DQ223728) overlapped only for 397 out of 658 bp with our haplotype, with two mismatches. Genetic distance between the two Chinese sequences was 1.07% (±0.005). The highest inter-group distance among *A*. *bungii* COI haplotypes resulted between Chinese sequences and Campanian ones (0.7%), while the lowest between German and Campanian haplotypes (0.4%).

**Table 2:** Localities and host species where RLB stages were collected. For each specimens COI and 28S-D2 were sequenced.

Sample	Localities	Province	Host	Year	Coordinates	Genbank Accession Code	
						COI	28S-D2
ABA1	Riserva Astroni	Naples	*P*. *armeniaca*	2013	40°50′N, 14°09′E	MN662926	MN658539
ABA2						MN662927	MN658540
ABA3	Via Cinthia		*P*. *domestica*	2013	40°50′N, 14°11′E	MN662928	MN658541
ABA4						MN662929	MN658542
ABA5	Via Cavone		*P*. *avium*	2017	40°52′N, 14°13′E	MN662930	MN658543
ABA6						MN662931	MN658544
ABA7			*P*. *domestica*	2017		MN662932	MN658545
ABA8						MN662933	MN658546
ABA9	Via Campana		*P*. *armeniaca*	2017	40°50′N, 14°07′E	MN662934	MN658547
ABA10						MN662935	MN820636

*Aromia bungii* samples collected in Campania also shared the 28S-D2 haplotype, which is identical to the only two present in GenBank (accession numbers HQ832606 and KF142125) whose origins are presumably Chinese.

### Fecundity tests

**Figure 1:**
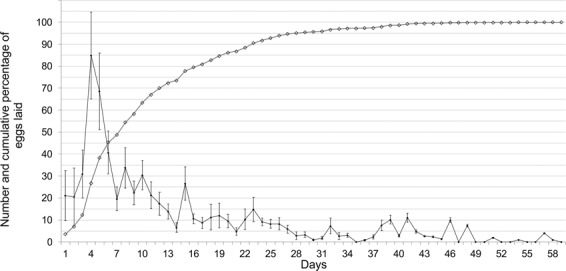
Daily distribution (mean ± SE) and cumulative percentage of eggs laid at 25 °C by *A*. *bungii* females.

**Figure 2:**
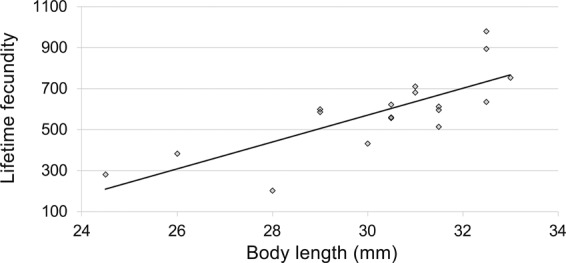
Regression between lifetime fecundity and body length in *A*. *bungii* ovipositing females (n = 18); Lifetime fecundity = −1392.85 + 65.4061*Body length; F = 24.91; df = 1, 16; *P* = 0.0001.

Females started oviposition 2.1 ± 0.18 days after their emergence (pre-oviposition period) and proceeded until the 58^th^ day. The mean OP was 22.1 ± 2.96 days. Daily distribution showed that the highest number of eggs laid per day was 84.8 ± 20 and was recorded on the 4^th^ day since emergence (Fig. 1). The mean LF was 587.5 ± 44.97 eggs and females laid 50 ± 4% of eggs in the first week after emergence, with the number of eggs laid per day decreased gradually in the following weeks (Fig. 1). Mean OR was 24.2 ± 3.51 eggs per day. Mean longevity of ovipositing females was 34.7 ± 2.74 days, and a weak relationship with LF was found (r = 0.195, F = 5.11; df = 1, 16; *P* = 0.038). Multiple regression analysis reported the following standardized regression equation: log_10_ LF = 0.0358 log_10_ OP + 0.0370 log_10_ OR. This indicates that OP and OR had almost the same effect on the LF. A moderately strong relationship was found between LF and female body length (Lifetime fecundity = −1392.85 + 65.4061*Body length; F = 24.91; df = 1, 16; *P* = 0.0001; r = 0.5844) (Fig. 2), while a relatively weak correlation (r = 0.192, F = 5.04; df = 1, 16; *P* = 0.039) resulted between female body length and longevity.

### Fertility and hatching period

**Figure 3:**
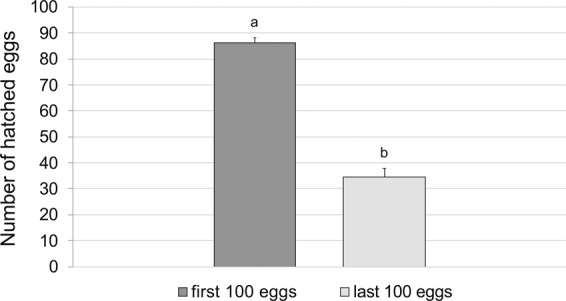
Comparison between the number of hatched eggs (mean + SE) of the first and the last 100 eggs laid by *A*. *bungii* (n = 18). Bars with different letters indicate significant difference at the 5% confidence level.

**Figure 4:**
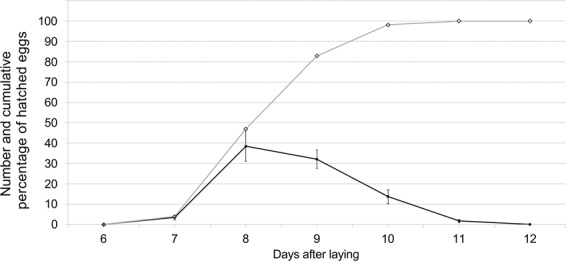
Hatching period (days after laying) and cumulative percentage of hatched eggs of *A*. *bungii* (mean + SE) at 25 °C (first 100 laid eggs).

The hatching rate was 75.6 ± 2.30% (range: 58.7–86.7%). Fertility was not correlated with body length (r = −0.058, F = 0.06, df = 1, 16; *P* = 0.81), longevity (r = −0.010, F = 0.84; df = 1, 16; *P* = 0.374) or LF (r = −0.053; F = 0.14; df = 1, 16; *P* = 0.71). Differences in hatching rate between the first and last 100 eggs laid showed that the earlier eggs were more fertile than the later ones (ANOVA, F = 312.27; df = 1, 34; *P* < 0.0001) (Fig. 3). Eggs hatched 8.7 ± 0.03 days after laying (range: 7–11 days). The highest number of eggs (38.6 ± 7.62%) hatched on the 8th day of oviposition (Fig. 4).

### Ovarioles number and ovigeny index

**Figure 5:**
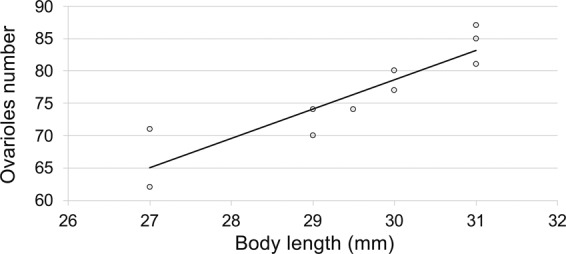
Regression between ovarioles and body length in newly emerged *A*. *bungii* females (n = 10); Ovarioles number = −57.2077 + 4.52658*Body length; F = 35.09; df = 1, 8; *P* = 0.0004.

The mean number of ovarioles per ovary was 76.1 ± 2.38 (n = 10) and ranged between 62 and 85. A relatively strong relationship was found between ovarioles number and female length (Ovarioles number = −57.2077 + 4.52658*Body length; F = 35.09; df = 1, 8; *P* = 0.0004; r = 0.7911) (Fig. 5). Newly eclosed females had a mean of 385.6 ± 22.74 mature eggs. The ratio between egg load at emergence and realized fecundity indicated that *A*. *bungii* is a slightly pro-ovigenic species. Ovigeny index was 0.66 ± 0.003.

### Adult longevity

**Figure 6:**
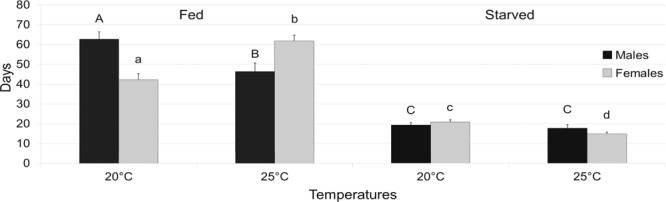
Longevity of fed (left) and starved (right) adults (♂ and ♀) of *A*. *bungii* (mean + SE) at two different temperatures (bars with different upper and lower case letter indicate significant difference at 5% confidence level).

Among adults that were provided with food, males lived significantly longer at 20 ± 1 °C (62.7 ± 3.75 days) than at 25 °C (ANOVA, F = 8.18; df = 1, 28; *P* = 0.008), while the longest non ovipositing female lifespan was recorded at 25 ± 1 °C (61.9 ± 2.81 days, n = 15) (ANOVA, F = 21.4; df = 1, 28; *P* = 0.0001) (Fig. 6). The latter lived significantly longer than ovipositing females (34.7 ± 2.74 days, n = 18) (ANOVA, F = 47.3; df = 1, 31; *P* < 0.0001) (Fig. 6). Starved females (20.9 ± 1.25 days) lived significantly longer at 20 ± 1 °C, than at 25 °C (ANOVA F = 15.51; df = 1, 28; *P* = 0.0005), while no difference was found between starved males at the two tested temperatures (ANOVA, F = 0.51; df = 1, 28; *P* = 0.48) (Fig. 6). A moderately strong relationship was found between body length and longevity for fed females at 25 ± 1 °C [r = 28.5, F = 6.6; df = 1, 13; *P* = 0.02], while no relationship was found for males [r = −0.004, F = 0.94; df = 1, 13; *P* = 0.35].

## Discussion

Multiple or single introduction of an invasive species can be discriminated by the identification of the haplotypes involved in the invasion process^36–38^. Molecular analysis of the Italian (southern Italy) specimens showed no haplotype variability; this could be due to the founder effect (reduced or no genetic variation that occurs when a population is established by a single or a few specimens)^39^. This suggests that *A*. *bungii* arrived in Italy by a single introduction event and with few individuals. This is a very common pattern found also in other invasive species recently recorded in Italy^40,41^.

The genetic variability of the *A*. *bungii* populations in the area of origin is not yet well defined, and the single haplotype found in Campania did not match either the only haplotype known from China nor the only haplotype found in Germany. Hence, to date we are not able to establish the region of origin of studied specimens, but we can speculate that the invasive process in Europe started from at least two independent introductions occurred in continental and peninsular Europe, respectively.

Knowledge of biological traits of invasive species is strategic to design the best control strategies. Colonisation and damage levels depend on several factors, especially on the reproductive potential of the invaders^42^. Sex ratio of *A*. *bungii* obtained in this study is slightly higher than the values reported by other authors (0.5 vs 0.43)^26^. All adults used in the present study were obtained from infested material collected in the field. In accordance with previous studies^5,43^, the mean adult body length was 31.2 and 29.2 mm for females (n = 93) and males (n = 65), respectively. Our estimate of mean lifetime fecundity of *A*. *bungii* is 587.5 eggs (ranging from 201 to 978), which is within the ranges reported in the Cerambycinae subfamily^44,45^. Previous observations carried out in China showed that RLB females can lay in average 324.6 to 357.0 eggs (ranging from 91 to 734), but the authors did not specify the number of replicates and pairs managements^26,35^. Pair management is very important and can strongly affect the number of laid eggs, because if adults are not regularly coupled, oviposition is reduced, as already reported for other species of cerambycids^46,47^. Investigations on LF in the genus *Aromia* are limited to *A*. *moschata* (Linneus 1758), the musk beetle. Duffy^48^ reported for this congeneric species an average number of eggs laid much lower than that recorded for RLB in this study. This is certainly due to the different size of the eggs belonging to the two species as compared by us. The volume of the egg of *A*. *moschata* is about five times that the one of *A*. *bungii* (Garonna, personal observation). Our estimates of *A*. *bungii* lifetime fecundity are also higher than those known for other wood-borer pests in the same subfamily, such as *Trirachys sartus* (Solsky, 1871) and *Osphranteria coerulescens* Redtenbacher, 1850^49,50^. Path analysis indicated that female body size expressed by body length affected positively the LF. A positive correlation between LF and female size is very common in longhorn beetles belonging to both Cerambycinae and Lamiinae^51^.

The number of RLB ovarioles per female was variable, ranging from 62 to 85. A positive correlation with body length indicated that long-sized females have a greater ovarian mass and egg productivity per ovariole compared with small females. Ovigeny index (0.66) showed that *A*. *bungii* is a slightly pro-ovigenic species, meaning that the female emerges with just over half of her eggs mature. This result is congruent with the record that some females laid the first eggs within 24 hours after emergence, with the short pre-oviposition period (2.1 days), and with the high number of eggs laid in the first week. So far, most of the longhorn beetles studied have been reported as synovigenic species, but the ovigeny pattern has been calculated through a different methodology (daily fecundity, pre-oviposition and oviposition period)^51–54^.

The oviposition period of *A*. *bungii* was highly variable among tested females ranging from 18 to 58 days and the mean value was about a month, similar to 26.3 days reported by Hu *et al*.^26^ and comparable to that of other longhorn beetles like the lamiine *Monochamus urussovii* (Fischer - Waldheim, 1806) and *Paraglenea fortunei* (Saunders, 1853)^53,55^. Conversely, OP was lower than that of cerambycine *Cerambyx welensii* Küster, 1846 and *Cerambyx cerdo* Linneus, 1758^51,54^. Mean OR averaged 22.1 eggs and was very close to the reported value in the subfamily Cerambycinae^51^. The highest mean number of laid eggs per day in *A*. *bungii* was 84.8 and the considerable oscillations during females LF, with inconsistent values ranging from 0 to 281 eggs per day, are common in longhorn beetles^51,54,56^ and often related to intraspecific differences in female size, oogenesis rate or ovarioles number^57^. Rate and period of oviposition had a similar effect on LF of *A*. *bungii*, differently from aforementioned lamiine species where the oviposition period contributed almost twice to the difference in lifetime fecundity than the rate of oviposition^53,55^.

About 75% of RLB eggs hatched and the mean egg developmental time was 8.7 days, at 25 °C. Previous laboratory studies on *A*. *bungii* have reported higher fertility values ranging between 94.3 and 97.6% and congruent values of egg developmental time (4 to 14 days)^26^. However, in the latter study, no data about the laboratory conditions of the studies, particularly temperatures, were provided. Abiotic factors, such as temperature, are known to be particularly crucial in affecting insect life history such as developmental period, longevity, fecundity, and fertility, as well as flight and behaviour^58^. In coleopteran species and, in particular, in longhorn beetles, it is widely demonstrated that temperatures strongly affect embryonic development and thus the egg developmental times^59–62^. Tests conducted at similar temperatures on the invasive species *Anoplophora glabripennis* (Motschulsky, 1853), showed lower fertility^61^ than those observed for *A*. *bungii* in the present study, while egg developmental time of RLB was similar to the cerambycine *Xylotrechus arvicola* Olivier, 1795^62^. The earlier eggs laid by RLB were more than twice as fertile as the later ones. Offspring rate production, indeed, is variable during insect lifetime; egg fertility decreases towards the end of the female lifespan^58,63,64^.

Fed females of *A*. *bungii* lived on average two months and about two weeks longer than males, at 25 °C. Longevity recorded in the present study is congruent with that of previous laboratory studies on cerambycids in which females usually lived longer than males and the mean adult longevity values ranged from about one month to more than seven months^65^. Conversely, at 20 °C, fed males lived longer than fed females, as reported also for the cerambycine *Phoracantha semipunctata* (Fabricius, 1775) and the lamiine *An*. *glabripennis*^61,66^. Not surprisingly, starved RLB adults lived two weeks less than fed at both tested temperatures, unlike the starved adults of the lamiine *Glenea cantor* (Fabricius, 1787) that died only a few days after emergence (2.47 and 4.71 days for males and females, respectively)^52^. In longhorn beetles, it is widely demonstrated that fed adults are more long-lived than starved^47^. Laying females used in fecundity tests showed a shorter lifespan than virgin females (about half), at 25 °C. This result confirms that the longevity of female decrease when individuals invest in the cost of reproduction, mating in particular^67,68^.

Information on the reproductive biology of exotic species is one of the requirements for successful eradication programs against invasive pests^42^. The data collected in this study suggests that a successful control strategy of RLB is hard to implement, and eradication measures need that all infested trees must be cut, chipped, and set on fire. Simultaneously, an early and correct flight monitoring of RLB adults is necessary for determining the best strategy to improve its management. Recent research on the efficacy of the identified pheromones of RLB^31,33,34^ has opened interesting monitoring and control scenarios (i.e., mass trapping) where outbreaks of the pests are recorded. Timing of insecticide treatments is another critical issue. Currently, a single active ingredient is registered in Italy for crop use against *A*. *bungii* (deltamethrin with three applications per year)^13^. This product may not be enough effective in killing emerging adults because of protracted emergence period and extended adult longevity. In addition, the short pre-oviposition period reduces the effectiveness of this control method. However, in an Integrated Pest Management (IPM) approach, additional practices must be combined, including physical, cultural and biological strategies. Experimental methods of biological control by entomopathogenic nematodes and fungi, already studied for other invasive wood-borer pests as *An*. *glabripennis* and *Anoplophora chinensis* (Forster, 1771)^7,22^, could be applied as containment measures to RLB to significantly reduce its biotic potential.

## Conclusion

This is the first detailed study on molecular and biological characterization of the invasive pest *A*. *bungii*, focusing on the basic aspects of the reproductive biology, in particular on its lifetime fecundity. All the specimens collected in Italy share the same mitochondrial haplotype, suggesting a single introduction or multiple introductions from the same source. Biological traits obtained show that RLB has a high biotic potential that can allow the beetle to establish, in a short time, a harmful population in a new area, if it will be accidentally introduced, as it happened in southern Italy and Japan. Biological data here presented will be useful to establish new action guidelines to contain the infestations of *A*. *bungii* in the outbreak areas, and to carry out an adequate IPM program in southern Italy. However, further in-depth studies are needed, in particular to clarify the duration of its still largely unknown life cycle.

## Methods

### Collection of RLB from infested logs

Adults and immature stages of *A*. *bungii* were obtained from infested plants in the outbreak area west of Naples (Campania Region, southern Italy, 40°50.423′N, 14°10.862′E and 40°50.340′N, 14°10.767′E) that were cut down during winter 2016–2017 under the eradication programme coordinated by the Regional Plant Health Service. The area included non-specialized orchards where stone fruits are predominant (especially *Prunus domestica*, *P*. *armeniaca*, and *P*. *avium*).

RLB infested trees were recognized by the presence of frass at tree base and along branches. The presence of typical exit holes and mature larvae during trunks inspection allowed the recovery/collection of samples to employ in the scheduled biological studies. Infested plants were cut and divided into 50–60 cm logs, sealed at both ends with a double layer of polyester nonwoven and moved to the laboratory. Trunks were stored in entomological cages and placed in a quarantine facility at 25 ± 1 °C, 60 ± 5% relative humidity (RH) and photoperiod 16 L:8D to allow adult emergence.

Emerged adults were daily collected and individually stored in plastic containers to prevent adults from injuring each other and randomly assigned to the different tests. For each adult, body length (from mandibles to anal pore along the ventral surface), elytra, and metatibia were measured. Few deformed or dying adults were discarded. Sex ratio was calculated following Kenneth and Hardy^69^. At the end of the tests, all wooden material was burned. Any adult beetles and larvae that remained alive were frozen at −20 °C.

### Morphological and molecular identification of RLB

Adults and mature larvae of *A*. *bungii* were identified following the taxonomic keys, descriptions and comparative images available in Matsushita^70^, Gressit^5^ and Duffy^24^. To characterize *A*. *bungii* and to estimate the genetic diversity of introduced populations, several samples were collected on different recorded host species in four localities (Table 2).

*Aromia bungii* DNA was extracted from single individuals listed in Table 2 through a slight modification of Chelex and proteinase K based method described in Gebiola *et al*.^71^. For each sample, a foreleg with small shreds of muscular tissue was separated with tweezers, soaked in a solution of 6 μl proteinase K (20 mg/ml) and 100 μl (5%) Chelex 100 (Bio-Rad, Richmond, CA) and incubated at 55 °C for 2 hours.

Extracted DNA was employed to amplify both a portion of the mitochondrial gene *Cytochrome c Oxidase* subunit I (COI) and the ribosomal gene 28S-D2 using primer pairs LCO/HCO^72^ and D2F/D2R^73^, respectively. PCR reactions and cycling conditions for COI and 28S-D2 were set as described in Gebiola *et al*.^71^.

PCR products were checked on a 1.2% agarose gel stained with GelRED (Biotium, Fremont, CA, USA) and sequenced. Chromatograms were assembled and edited by eye with Bioedit 7.2.5^74^. Edited COI sequences were virtually translated into the corresponding amino acid chain to detect frame-shift mutations and stop codons, using EMBOSS Transeq [http://www.ebi.ac.uk/Tools/st/emboss_transeq/(accessed 10 sep 2019)]. Edited sequences were checked against the GenBank database by Blast nucleotide searches and were submitted to the GenBank database with accession numbers as in Table 2. COI haplotypes distances and standard errors were calculated with MEGA 6 software^75^ as “uncorrected *p*-distance” including homologous sequences of RLB available in GenBank (accessed 13 Sep 2019).

### Fecundity tests

RLB lifetime fecundity (LF) was assessed by using 18 females. Fresh and uninfested *P*. *armeniaca* branches with regular bark and free from protruding twigs were selected to obtain logs with a mean length of 35 cm and a mean diameter of 7 cm. Newly emerged females were allowed to mate with a male for about two hours and then placed in a transparent plastic container (45 × 30 × 25 cm) covered by a rigid tulle fabric. Each female was daily fed with a fresh apple piece and a new log was placed in the container to allow oviposition until female death. Weekly, a male was placed for 24 h in the same container for mating. Every 24 h all logs were examined under a binocular microscope at X 40 magnification to count the eggs laid and then kept at 25 ± 1 °C until egg hatching. The longevity of ovipositing females was recorded. We determined the following variables for each female: 1) lifetime fecundity (LF = total number of eggs laid during her lifetime); 2) oviposition period (OP = the time elapsed between the first and last egg laid); and 3) oviposition rate (OR = mean number of eggs laid per day during the OP, i.e., OR = LF/OP) as proposed by Togashi and Yamashita^53^. Correlations between body size, longevity, and LF of ovipositing females were assessed.

### Fertility and hatching period

Fertility was calculated as the hatching rate of laid eggs by single female used in the fecundity tests^58^. Logs exposed 24 h to RLB adults were observed daily. Hatched eggs were labeled each day with a different color and hatching rate was recorded. Eggs were considered hatched when newborn larvae penetrated the log bark and light frass was produced around the oviposition site. The fertility of the first and last 100 eggs laid by each female were compared. The hatching period (i.e., days after deposition) was assessed on the first 100 eggs laid by each female.

### Ovarioles number and ovigeny index

Numbers of ovarioles and full size eggs per female were estimated by dissecting females within 24h after emergence in 0.8% saline solution (n = 10). The ovigeny index was calculated by dividing the initial mean load of mature egg by the maximum average lifetime fecundity (i.e., realized fecundity *sensu*)^76^.

### Adult longevity

Adult longevity was evaluated on fed and starved adults (n = 15) at two different temperatures (20 and 25 ± 1 °C) with photoperiod 16 L:8D and RH of 60 ± 5%. Newly emerged adults were singly isolated in polyethylene bottle (bottom diameter 67, top diameter 50 and height 90 mm with a tulle cover) and assigned to one of the temperature treatments. Adults were daily provided with apple pieces as fresh food and checked for mortality. After death, elytra, metatibia, and body length were measured on each tested adult. The possible correlation between longevity and body size was evaluated only on fed males and females at 25 ± 1 °C. To evaluate the effect of oviposition on lifespan, the longevity of fed females at 25 ± 1 °C was compared with that of fed females used in the fecundity test.

### Statistical analysis

Data satisfying conditions of normality and homoscedasticity, both untransformed or after appropriate transformation (cosine transformed, in the comparison between the first 100 and the last 100 eggs laid), were analysed by ANOVA and the means were separated at the 0.05 level of significance by a multiple range test (Tukey HSD or, in case of unequal samples, Bonferroni)^77^. All relationships between variables were assessed by regression analysis. To determine the effects of OP and OR on differences in lifetime fecundity among females, a multiple regression analysis was performed to obtain the standardized partial regression coefficients of log_10_ OP and log_10_ OR. All data are presented non-transformed, with standard error within brackets.
